# Isolated human lens interferometric surface radius of curvatures: Implications for the mechanism of accommodation

**DOI:** 10.1371/journal.pone.0327028

**Published:** 2025-06-24

**Authors:** Ronald A. Schachar, Ira H. Schachar, John Fabio, Dani Balicki, Nabeel Sufi, Barbara K. Pierscionek, Boyd Hunter

**Affiliations:** 1 Schachar LLC, La Jolla, California, United States of America; 2 North Bay Vitreoretinal Consultants, Santa Rosa, California, United States of America; 3 Zygo/Ametek, Tucson, Arizona, United States of America; 4 Faculty of Health, Medicine and Social Care, Medical Technology Research Centre Anglia, Ruskin University, Chelmsford, United Kingdom; 5 Praxis Optics, Elmira, New York, United States of America; National Eye Institute, UNITED STATES OF AMERICA

## Abstract

Accurate central surface radius of curvature (RoC) measurements of isolated human lenses are essential for understanding the zonular forces required to modify human lens shape to focus at near; i.e., accommodate. The human lens can be described as an encapsulated oblate spheroid, with its minor axis aligned with its optical axis. The lens is suspended by zonular fibers that originate from the epithelium of the ciliary body and insert into the equatorial region of the lens capsule. According to Helmholtz’s theory of accommodation when the eye views a distant object (the unaccommodated state), the ciliary muscle is fully relaxed and the zonules are under maximal tension. This tension flattens both the central and peripheral lens surfaces resulting in minimal central optical power (COP). During near focus (accommodation), contraction of the ciliary muscle reduces zonular tension, allowing the elastic capsule to restore the lens to a more rounded shape. This increases the curvature of the lens surfaces, central thickness, and COP. Consequently, isolated lenses without zonular tension from young donors (20–30 years old) would be expected to exhibit maximum COP. However, the companion independent profilometric equation fitting study found that, within central optical zones ≤ 3 mm, 10 fresh isolated lenses from donors in this age range actually had minimal COP. The present study utilizes a white light scanning interferometer (WLSI) with a 10x objective that was validated by measuring RoCs of glass and porcine lenses. Fourteen transparent human lenses were obtained from both eyes of seven donors aged 20–30 years of whom 2 were female and 5 were male. One lens of each donor was placed in preservative media and the contralateral lens in culture media within 11:26 ± 5:15 (range: 4:47–21:54) of the donor’s death. Two of the lenses stored in the culture media had torn capsules and were excluded from the study. Central thickness and WLSI surface vertex RoCs of 12 lenses were measured within 16:27 ± 5:22 (range: 10:11–25:33) of the donor’s death. Mean central thickness, anterior and posterior vertex RoCs and COP were 3.54 ± 0.07 mm, 10.2 ± 0.9 mm, 6.8 ± 1.0 mm, and 20.7 ± 2.1 diopters, respectively. These results confirm the companion study that isolated human lenses have low COP consistent with the unaccommodated state of lenses *in vivo*. Therefore, relaxation of all the zonules does not increase COP and cannot be the basis for the mechanism of accommodation. These results have implications for the development and treatment of myopia, presbyopia, glaucoma, cortical cataracts and design of accommodative intraocular lenses.

## Introduction

In 1801, Thomas Young definitively demonstrated that the increase in central optical power (COP) of the human eye required for near vision (accommodation) is solely due to a change in the shape of the lens [1]. The human lens is supported 360 degrees by anterior, equatorial and posterior zonules. The anterior and posterior zonules are approximately 150 microns in diameter while the equatorial zonules are only 15 microns in diameter. The equatorial zonules, which are present throughout life, originate in the valleys between the ciliary processes and insert into the lens capsule at the lens equator. The anterior and posterior zonules originate at the pars plana and proceed anteriorly while being held adjacent to the surface of the ciliary body by tensor zonules. The posterior zonules separate from the ciliary body prior to the anterior zonules, and the anterior and posterior zonules insert into the lens capsule anterior and posterior to the lens equator, respectively [2–5].

Two major theories have been proposed to explain the mechanism of accommodation. Both agree that all the zonular fibers are under tension when the eye is focused at a far distant object (the unaccommodated state). However, the Helmholtz theory [6] postulates that in this state the ciliary muscle is relaxed and all the zonules are under maximum tension. In contrast, Schachar hypothesizes that in the unaccommodated state while the ciliary muscle is fully relaxed, all the zonules are under the minimum tension necessary to maintain lens stability [7–9].

According to Helmholtz, during accommodation ciliary muscle contraction causes a reduction in tension of all zonular fibers allowing the elastic lens capsule to round up the lens, which leads to a decreased equatorial diameter, increased central thickness and increased COP. Consequently, it has been concluded that isolated human lenses without zonular tension have maximum COP and are equivalent to *in vivo* lenses during maximum accommodation [10–14].

Alternatively, during accommodation, Schachar proposes distinct actions of the three different groups of zonules. Schachar believes that during accommodation, equatorial zonular tension increases while simultaneously anterior and posterior zonular tension decrease. The selective increase in equatorial zonular tension results from the unique orientation of the different ciliary muscle fiber groups. The increase in equatorial zonular tension is due to outward notching of the anterior ciliary muscle fibers [7,8,15–17]. The simultaneous decrease in anterior and posterior zonular tension is due to forward movement of the pars plana from contraction of the posterior longitudinal and posterior radial ciliary muscle fibers. Given the human lens is an encapsulated ellipsoid with negligible incompressible stroma and an aspect ratio (minor axis/major axis) ≤ 0.6, an increase in equatorial zonular tension leads to peripheral surface flattening, and counterintuitively, to steepening of the central surfaces with an increase in central lens thickness and COP [7,8,18–22]. This topography occurs to minimize changes in surface curvatures [7,18]. Schachar’s theory would anticipate that with zonular removal, the COP of the isolated lens would be minimal and diametrically opposite to the high lens COP expected with the Helmholtz hypothesis.

Multiple studies support Helmholtz’s theory that the isolated lens has maximum COP by evaluating surface radius of curvatures (RoCs) at central optical zones ≥ 4 mm diameter or by measuring lenses that were not fresh [10–14]. However, the companion study found that the COP at central optical zones ≤ 3 mm from shadowgraph profilometer measurements of fresh isolated human lenses from young donors (20–30 years old) were low [23]. This finding was consistent with studies that measured RoCs of isolated human lenses by phakometry [6,24,25] and lens topography [26].

To resolve the diametrically opposed results of these studies, objective accurate lens surface RoCs measurements of fresh isolated human lenses without zonular tension are required and are fundamental for understanding the mechanism of accommodation. Curve fitting and shadowgraph profilometer RoC measurements are subjective and have significant variability. To overcome these deficiencies, white light scanning interferometry (WLSI) was utilized in the present study to objectively and automatically measure lens surface RoCs.

WLSI involves vertically scanning the lens surface reflected focal plane while acquiring multiple interference patterns with a reference surface. Then distances are determined from the association between phase variation and wavenumber. By analyzing the frequency domain, the WLSI lens surface RoCs are obtained while avoiding problems associated with variation in the source emission spectrum, distortion of the fringe contrast envelope and changes in the sampling interval [27–31].

We validated the WLSI by assessing RoCs of glass lenses. The glass lenses were measured dry and after a drop of saline was applied to the lens surface to emulate the wet surfaces of porcine and human lenses. Since the porcine lens has been a model for understanding the physical and physiochemical attributes of the human lens [32–35] and has been measured interferometrically [36,37], it provided the methodological basis for assessing the WLSI surface RoCs of isolated human lenses.

## Methods

### Glass lenses

The accuracy of the WLSI with a 10x objective (NewView, Zygo/Ametek, Tucson, AZ, USA) were evaluated by measuring the RoCs of uncoated double-convex glass lenses. The lenses had RoCs of 3.50, 6.58 mm, 7.59 mm, central thicknesses of 2.30 mm, 2.60 mm, 2.54 mm and clear apertures of 2.50 mm, 4.05 mm, 5.40 mm, respectively. Focal length and central thickness tolerances were 1% and 0.050 mm, respectively (stock #s 32-023, 45-176 and 32-021, Edmund Optics, Barrington, NJ, USA).

Using a linear beam of a red laser that was attached to a micrometer, central lens thickness was measured. Then the lens was placed on a rotational stage that was mounted on the WLSI so that the glass lens optic axis was in line with the WLSI objective. After this alignment, the WLSI automatically measured the vertex RoC and the RoCs when the lens was tilted 3°, 6°, and 9°. To emulate the wet surfaces of porcine and human lenses, soapy distilled water was applied to the glass lens surfaces and the measurements repeated. The soap was added to the saline to ensure it spread evenly over the glass surface and the liquid film was identified interferometrically. Due to the small diameter and steep curvature, angular rotation measurements of the glass lenses could not be obtained beyond 9°.

### Porcine lenses

Eyes from 6 month to 9 month old porcine donors were enucleated at a slaughterhouse within 1 hour of their death, placed on ice, and shipped to our laboratory in two separate lots one week apart. The porcine lenses were carefully removed from the enucleated eyes within 24 hours postmortem. To evaluate the reliability of the WLSI, the vertex ROCs of the anterior and posterior surfaces of three porcine lenses of lot # 1were each measured 10 times. The lenses were initially placed anterior surface up in a central concavity of an aluminum plate that was mounted on a tilting stage. The concavity had a 6.5 mm RoC. The optic axes of the porcine lens and the WLSI objective were aligned with the tilting stage at 0°. The WLSI then automatically found the lens vertex and determined the ROC. The lens was turned over and the posterior surface was measured.

A week later, central thickness and vertex RoCs of four porcine lenses from lot # 2 were measured. Central thickness was only measured on Day 1 to keep lens handling to a minimum. Following the measurements, two lenses were placed in separate sterile vials (Wheaton CryoElite, DWK Life Sciences, NJ, USA) containing 2.5 mL of Optisol (50006-OPT, Optisol-GS, Bausch & Lomb, Rochester, NY, USA) [26] and the 2 were placed in separated vials containing 2.5 mL of DMEM (D6421, Dulbecco’s Modified Eagle’s Medium/Nutrient Mixture F-12 Ham, Sigma-Aldrich, St. Louis, MO, USA) [38]. The lenses were stored overnight at 4° C. The vertex ROCs were measured and repeated daily for the next three days. After each daily measurement, the lens was placed in a new sterile vial containing fresh media. All measurements were made at room temperature and the RoC measurements of both lens surfaces were completed within less than 30 minutes.

### Human lenses

Following written informed consent, the San Diego Eye Bank procured the lenses that were evaluated in the present study. The San Diego Eye Bank (San Diego, CA, USA) is authorized for organ and/or tissue donation for research in accordance with the California‘s Uniform Anatomical Gift Act effective since 10-Aug-2020. For the present study, 14 lenses from seven unidentifiable donors aged 20–30 years were supplied from 30-Nov-2024–30-Mar-2025. There were 2 female and 5 male donors. One lens of each donor was placed in Optisol and the contralateral in DMEM within a mean of 11:26 ± 5:15 (range: 4:47–21:54) of the donor’s death and shipped on ice. This ensured that the lenses were as fresh as logistically possible. The 14 lenses were transparent without evidence of cataracts. Two of the lenses that had been placed in DMEM, one from a 21 y/o and the other from a 25 y/o donor, had torn capsules and were excluded from the study. Donor demographics, cause of death, and medical history are summarized in Table 1. Aside from three donors wearing spectacles, no significant ophthalmic history was reported.

**Table 1:** Demographics and medical characteristics of the donors.

Age (years)	Ethnicity	Gender	Cause of Death	Medical History
**20**	White	Female	Anoxia 2^nd^ to suicidal hanging	Pneumonia marijuana/tobacco use, anxiety
**21**	White	Male	Motorcycle accident, cardia arrest, subarachnoid hemorrhage, pneumothorax, chest fractures	No significant medical history
**23** ^a^	White	Male	Subarachnoid hemorrhage, anoxia	Headaches, seizures, polysubstance abuse, asthma, respiratory syncytial virus, pneumonia
**25**	White	Female	Cardiac arrest anoxic brain injury, drug overdose	Substance abuse
**27** ^a^	Hispanic	Male	Metastatic bladder cancer	Lower extremity paralysis 2^nd^ to cancer, alcohol use
**29**	White	Male	Anoxia	Type 2 diabetes, morbid obesity, hyperlipidemia
**30** ^a^	White	Male	Ventricular fibrillation, cardiac arrest	Congenital heart defects, stents, heart valve replacement, type 2 diabetes mellitus, tobacco/alcohol use, asthma, lower extremity blood clot

^a^Donor wore spectacles.

Central thickness and WLSI surface vertex RoCs of the 12 lenses were measured within 16:28 ± 5:22 (range: 10:11–25:33) of the donor’s death. All vertex RoC measurements were first made with the anterior lens surface up and then the lenses were turned over to measure the posterior surface. To minimize variability, ROC measurements were only performed when the liquid film thickness on the lens surface was less than 3 microns.

Central lens thickness and WLSI RoC measurements were performed at room temperature following the same procedures as the porcine lens. COP was calculated with the following thick lens formula [39]:


$$\begin{array}{c} COP\;(diopters)=\frac{n_{l}-n_{a}}{r_{a}}+\frac{n_{a}-n_{l}}{r_{p}}-\frac{t\left(n_{l}-n_{a}\right)\left(n_{a}-n_{l}\right)}{n_{l}r_{a}r_{p}} \end{array}$$ (1)

where *r*_*a* _ = anterior lens surface RoC, *r*_*p*_ = posterior lens RoC (negative by convention), *t *= central lens thickness, $\backslash (n_{a\;}\backslash )$= 1.336 and $\backslash (n_{l\;}\backslash )$= 1.42 were the refractive indices of aqueous humor/vitreous and lens, respectively.

**Fig 1:**
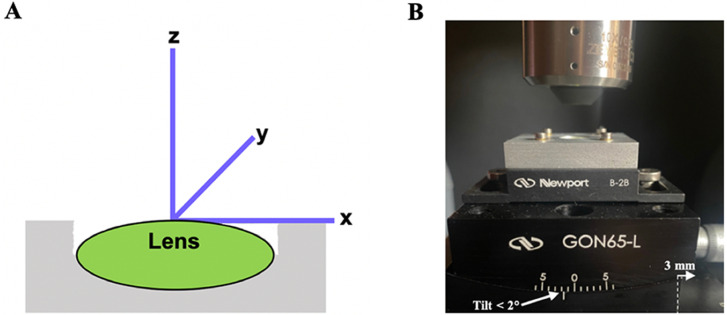
Schematic of the WLSI coordinate systems. To assess peripheral RoCs, A) the lens was initially tilted counterclockwise around the y-axis in 2° steps from 0° to 20°; however, the lens shifted from its baseline position as the angle was increased. **B)** Even when a goniometer was used so that < 2° of tilt was required, the lens shifted precluding the measurement of peripheral RoCs.

For eight lenses, 10 consecutive vertex RoCs of the anterior and posterior lens surfaces were interferometrically measured. For the subsequent four lenses, peripheral RoCs were targeted by incrementally tilting the rotational stage counterclockwise about the y-axis in 2° steps, ranging from 0° to 20°. However, with increasing tilt angle, each lens exhibited positional drift relative to its initial alignment, thereby limiting the measurements to vertex RoCs only. Even when a goniometer with a long 101.6 mm axis of rotation was used (Model: GON65-L, MKS/Newport Andover, MA, USA) so that < 2° of tilt was required, the lenses still shifted precluding the measurement of peripheral RoCs (Fig 1).

## Results

### Glass lenses

**Fig 2:**
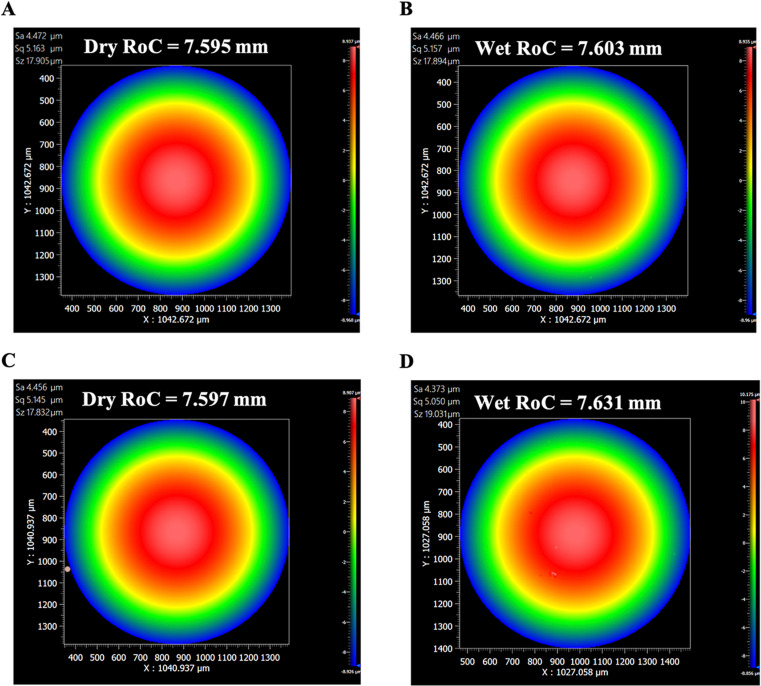
Interferometric images of the RoC = 7.59 mm glass lens at 0° and 9° tilt when the lens was dry A) and C) RoC measured 7.595 mm and 7.597 mm, and when wet B) and D) RoC measured 7.603 mm and 7.631 mm, respectively. The abbreviations Sa, Sq and Sz are surface roughness parameters for the measured area. Sa = absolute mean height difference of each point from the mean lens surface height, Sq = root mean square height and Sz = maximum height [40].

Measured mean SD central thickness for the 3.50, 6.58 and 7.59 glass lenses were 2.32 ± 0.03 mm, 2.62 ± 0.03 mm and 2.56 ± 0.02 mm. The maximum difference between the interferometrically measured mean RoCs and the reported RoCs for all the lenses when dry was < 10 μm and when wet was < 20 μm, which was within the manufacturer’s reported tolerance. Independent of the angular rotation of the glass lenses from 0° to 9°, there was no statistically significant difference in the measured RoCs between the wet and dry lenses as shown in Table 2 and Fig 2.

**Table 2:** Interferometric dry and wet glass lens RoCs.

Reported RoC (mm)	Tilt Stage Angle (degrees)	Dry RoC (mm)	Wet RoC (mm)	Absolute Difference |Wet RoC- Dry RoC| (mm)	p-value^a^
**7.59**	0.0	7.595	7.603	0.008	**0.06**
	3.0	7.601	7.621	0.020	
	6.0	7.599	7.608	0.009	
	9.0	7.597	7.631	0.034	
	**Mean**	**7.598**	**7.616**	**0.018**	
	**SD**	0.003	0.013	0.012	
**6.58**	0.0	6.582	6.589	0.007	**0.22**
	3.0	6.584	6.556	0.028	
	6.0	6.585	6.574	0.011	
	9.0	6.589	6.577	0.012	
	**Mean**	**6.585**	**6.574**	**0.015**	
	**SD**	0.003	0.014	0.009	
**3.50**	0.0	3.504	3.505	0.001	**0.13**
	3.0	3.482	3.489	0.007	
	6.0	3.489	3.497	0.008	
	9.0	3.499	NA	NA	
	**Mean**	**3.494**	**3.497**	**0.005**	
	**SD**	0.010	0.008	0.004	

^a^Two-sided paired Student’s t-test for the difference between the wet and dry glass lenses.

NA = not applicable since the liquid rolled off the surface of the glass lens at 9° tilt.

### Porcine lenses

**Fig 3:**
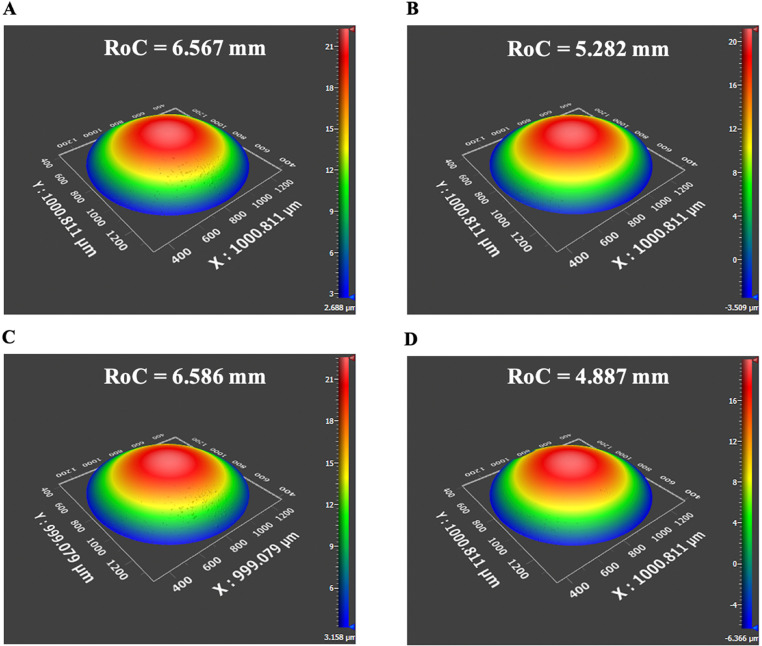
Interferometric porcine lens RoCs after 1 day of refrigeration at 4° C in Optisol for the A) anterior surface and B) posterior surface, and in DMEM for the C) anterior surface and D) posterior surface.

**Fig 4:**
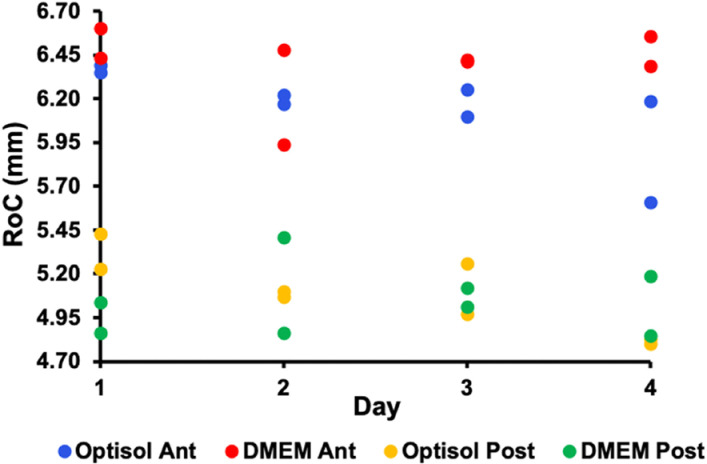
RoC vs time for the four lenses of lot # 2. There was no meaningful difference in the anterior (Ant) or posterior (Post) surface RoCs between the lenses stored in Optisol and DMEM.

The vertex RoCs of each of the 3 lenses from lot # 1 for the anterior surface were 6.489 ± 0.018 mm, 6.580 ± 0.009 mm and 6.544 ± 0.046 mm and for the posterior surface 5.247 ± 0.003 mm, 5.383 ± 0.039 mm and 5.299 ± 0.027 mm. The total mean vertex ROCs for the lot # 1 lenses were 6.544 ± 0.046 and 5.311 ± 0.069 mm. For the lenses from lot # 2 on Day 1 stored in Optisol and DMEM the anterior surface vertex RoCs were 6.371 ± 0.029 mm and 6.516 ± 0.118 mm, and for the posterior surface 5.331 ± 0.137 mm and 5.135 ± 0.384 mm. The mean central thickness of the 4 lenses from lot # 2 on Day 1 was 7.19 ± 0.04 mm. This central thickness and the RoCs were consistent with published data [36,37]. Interferometric RoCs images of the lenses stored in Optisol and in DMEM are shown in Fig 3. There was no meaningful difference in the surface RoCs over the 4 days between lenses stored in Optisol and DMEM (Fig 4).

### Human lenses

**Fig 5:**
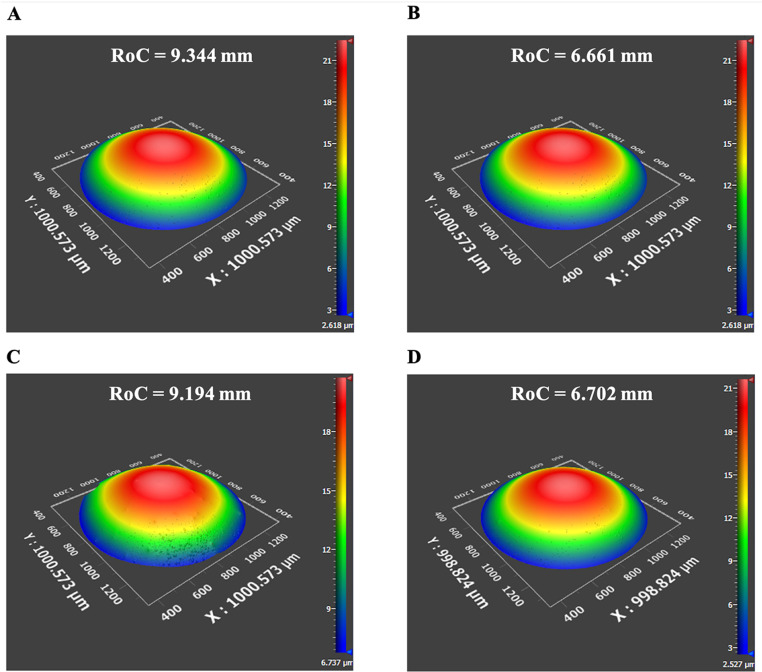
Interferometric RoCs of both human isolated lenses from a donor aged 29 years. Vertex anterior and posterior RoCs of the lens stored in Optisol (A and B) and the lens stored in DMEM **(C and D)**, respectively.

**Fig 6:**
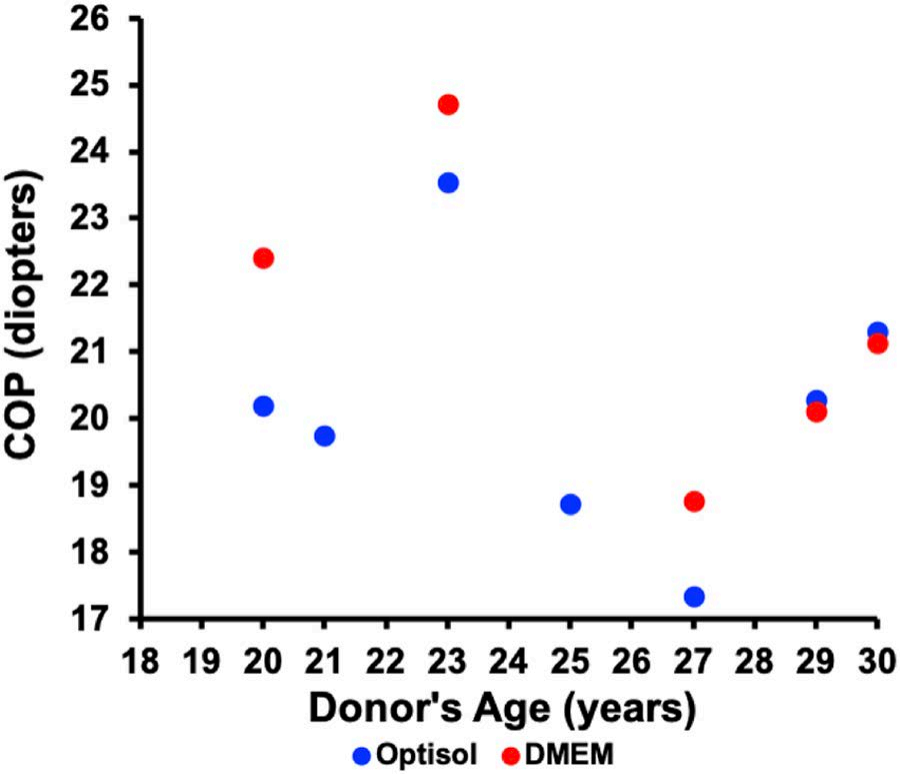
Calculated COP of isolated human lenses vs donor’s age. There was no statistically significant difference in COP between the lenses stored in Optisol and DMEM.

Interferometric vertex RoCs measurements were made within 16:28 ± 5:22 (range: 10:10–25:33) of the donor’s death. The central thickness, vertex anterior and posterior RoCs and calculated COP of the lenses were 3.54 ± 0.07 mm, 10.2 ± 0.9 mm, 6.8 ± 1.0 mm, and 20.7 ± 2.1 diopters, respectively (Table 3, Figs 5 and 6). There was no statistically significant difference between lenses stored in Optisol and DMEM, see Table 4. In Table 5, COP of the isolated lenses from the present study and the companion profilometric equation fitting study [23] were compared to accommodated and unaccommodated lenses *in vivo* [23,41–43]. In addition, the mean thickness of the isolated lenses (3.54 ± 0.07 mm) was nearly identical to the *in vivo* mean unaccommodated lens thickness (3.53 ± 0.09 mm) of age-matched individuals [23,41,43].

**Table 3:** Isolated human lens interferometric RoCs and calculated COPs.

Age (years)	Preservative Media	Thickness (mm)	Anterior RoC (mm)	Posterior RoC (mm)	COP (diopters)
20	Optisol	3.42	10.4	6.8	20.2
	DMEM	3.45	11.8	5.4	22.4
21	Optisol	3.50	9.1 ± 0.5^a^	7.8 ± 0.3^a^	19.8
23	Optisol	3.51	8.7	5.9	23.6
	DMEM	3.49	9.1	5.3	24.7
25	Optisol	3.54	9.8 ± 0.6^a^	8.1 ± 0.3^a^	18.7
27	Optisol	3.59	11.0 ± 0.3^a^	8.5 ± 0.1^a^	17.5
	DMEM	3.57	11.0 ± 0.7^a^	7.4 ± 0.6^a^	19.1
29	Optisol	3.62	10.5 ± 0.3^a^	6.7 ± 0.1^a^	20.3
	DMEM	3.57	10.2 ± 0.2^a^	6.7 ± 0.1^a^	20.2
30	Optisol	3.58	9.7 ± 0.4^a^	6.5 ± 0.1^a^	21.3
	DMEM	3.60	10.7 ± 0.4^a^	6.9 ± 0.1^a^	21.1
	**Mean**	**3.54**	**10.2**	**6.8**	**20.7**
	SD	0.07	0.9	1.0	2.1

^a^Mean ± SD of 10 interferometric RoC measurements.

**Table 4:** Statistical comparison of the paired lenses stored in Optisol and DMEM.

Age	Thickness (mm)		Anterior RoC (mm)		Posterior RoC (mm)		COP (diopters)	
	Optisol	DMEM	Optisol	DMEM	Optisol	DMEM	Optisol	DMEM
**20**	3.42	3.45	10.4	11.8	6.8	5.4	20.2	19.4
**23**	3.51	3.49	8.7	9.1	5.9	5.3	23.6	19.2
**27**	3.59	3.57	11.0	11.0	8.5	7.4	17.3	18.8
**29**	3.62	3.62	10.5	10.2	6.7	6.9	20.3	20.2
**30**	3.58	3.60	9.7	10.7	6.5	6.2	21.3	21.1
**p-value** ^a^	**0.85**		**0.19**		**0.08**		**0.12**	

^a^Two-sided paired Student’s t-test.

**Table 5:** Comparison of the isolated lenses to *in vivo* lenses.

Age	*In Vivo*			*In Vitro*	
	AA^a^	Accommodated^b^	Unaccommodated^b^	Mean of 4 Fitting Equations	Present Study
**20**	9.7	31.6	21.0	22.5	21.3^c^
**21**	9.4	31.2	21.1	23.8	19.8
**23**	8.9	31.1	21.2		24.2^c^
**25**	8.4	30.1	21.3	20.3	18.7
**27**	7.9	29.5	21.4	17.5	18.3^c^
**29**	7.3	28.9	21.5	21.0	20.3^c^
**30**	7.1	28.7	21.6	19.8	21.2^c^
**Mean**	**8.4**	**30.2**	**21.3**	**20.8**	**20.5**
**SD**	1.0	1.2	0.2	2.2	2.0

^a^Lower limit of accommodative amplitude [44].

^b^*In vivo* from Dubbelman et al. [23,41–43].

^c^Mean COP of lenses from the same donor that were stored in either Optisol or DMEN.

## Discussion

The widely accepted Helmholtz theory of accommodation is founded on the principle that an increase in COP results from a reduction in zonular tension. Accordingly, isolated lenses without zonular tension are expected to exhibit their maximum COP [6]. However, Helmholtz’s ophthalmometer measurements of *in vitro* lenses from donors aged 25–30 years revealed anterior and posterior surface RoCs of 10.2 mm, 8.9 mm, and 5.86 mm, 5.89 mm, respectively. These measurements agreed with his *in vivo* measurements of unaccommodated lenses, paradoxically contradicting his own theory. Furthermore, they correspond with anterior surface RoCs of 11.4 mm and 12.4 mm reported by Stradfelt [24] and Tscherning [25], respectively, as well as Schachar’s topographically measured anterior and posterior RoCs of 10.5 mm and 7.1 mm [26].

The present WLSI interferometric measurements automatically and objectively determined the RoCs of human isolated lenses from donors aged 20–30 years. All lenses were placed in preservative or culture media and measurements initiated within a mean of 11:26 ± 5:15 (maximum 21:54) and 16:28 ± 5:22 (maximum of 25:13) of the donor’s death, respectively. This ensured that the measurements were made on fresh lenses. There was no statistically significant difference in the vertex RoCs of the human lenses stored in preservative or culture media. The resulting mean anterior and posterior RoCs and calculated COPs were 10.2 ± 0.9 mm, 6.8 ± 1.1 mm, and 20.7 ± 2.1 diopters, respectively. These values are consistent with RoCs and COPs of central optical zones (3 mm diameter) obtained from curve fitting shadow profilometer x-y coordinates of fresh lenses obtained from donors in the same age range as noted in the companion paper [23]. Furthermore, these values are consistent with eleven *in vivo* studies of unaccommodated subjects aged 18–35 years (n = 117) that measured both anterior and posterior RoCs by optical coherent topography (OCT), Scheimpflug images, phakometry, magnetic resonance imaging (MRI) and/or Bessel beam phakometry [45–55]. The mean anterior and posterior RoCs from these *in vivo* studies were 11.79 ± 0.90 mm and 6.29 ± 0.26 mm, which are within 2 standard deviations of the means of the present study.

The lens surfaces were measured with the opposite surface resting on a firm support. A previous study demonstrated that when isolated lenses were floated, the mean RoC increased by 0.26 ± 0.21 mm (p < 0.01) relative to when the opposite surface was supported [26]. Accordingly, if the measurement approach used in the present study introduced any bias, it would have been toward an underestimation of RoC. Thus, the true RoC may be slightly greater than reported, further reinforcing the conclusions of the present study.

Interferometric measurements of the lens peripheral RoCs were not possible with the WLSI in the present study, which precluded assessment of spherical aberration of the isolated human lens. In the future, peripheral RoCs and accurate surface profiles of the human isolated lens may be evaluated by multi-wavelength interferometry [56] or multi-directional orthogonal lateral shearing interferometry [57].

Together, the present and companion profilometric equation fitting studies evaluated a total of 22 fresh isolated human lenses, without zonular tension, obtained from 17 donors aged 20–30 years. This donor age range was specially selected for their large accommodative amplitude [44]. These findings clearly demonstrate that the COP of isolated lenses align with the minimum COP of unaccommodated lenses *in vivo*. Therefore, relaxation of all the zonules cannot account for the mechanism of accommodation, thereby refuting the widely accepted Helmholtz theory [6]. In addition to this fundamental inconsistency, the Helmholtz theory proposes that zonular tension should be maximum when the eye is unaccommodated and that during accommodation both the peripheral and central lens surfaces steepen resulting in a shift of spherical aberration in the positive direction. However, zonular tension in the unaccommodated state is low [58], and during accommodation, the peripheral lens surfaces flatten [25,41] with spherical aberration universally shifting in the negative direction [59]. Moreover, finite element analysis demonstrates that for accommodation, the Helmholtz theory would require a total zonular force exceeding 0.05 N, which is greater than the ciliary muscle can exert [9,60].

In contrast, the Schachar mechanism predicts that in the unaccommodated state, zonular force is minimal, but sufficient to maintain lens stability as demonstrated by finite element analysis [9]. During accommodation, equatorial zonular tension increases while anterior and posterior zonular tension simultaneously decreases, maintaining lens stability. Maximum accommodation requires an equatorial zonular force of 0.020N [9]. The increase in equatorial zonular tension causes peripheral surfaces to flatten, thereby shifting spherical aberration in the negative direction. Recognizing that accommodation involves increased equatorial zonular tension necessitates a reevaluation of the pathogenesis and treatment strategies for myopia, glaucoma, presbyopia, and cortical cataracts, as well as the design of accommodative intraocular lenses.

## Supporting information

S1 Checklist PLOS One human subjects research checklist. (DOCX)
