# Supplementary material for: Isolated human lens interferometric surface radius of curvatures: Implications for the mechanism of accommodation
Source: PLoS One. 2025 Jun 24;20(6):e0327028. doi: 10.1371/journal.pone.0327028 (PMC12186931; doi:10.1371/journal.pone.0327028)
Supplement: S1 Checklist — (DOCX) [file pone.0327028.s001.docx]

**Human Participants Research Checklist**

***Complete the following if your study involved human participants or human participants’ data. These questions should be addressed for prospective and retrospective studies.***

1. Did you obtain ethics approval for this study?
   - If yes, please upload (file type “Other”) all the approval documents you received from your ethics committee to cover the entire range of the study period (i.e. the original approval document and any extension documents). Where ethics approval was obtained from more than one study location, please provide approval document(s) from all of the sites. If the original document is in another language, please also provide an English translation.

___ Uploaded **X** N/A

- - If you did not obtain ethical approval, please explain why this was not required below.

Following written informed written consent, the San Diego Eye Bank procured the lenses that were evaluated in the present study. The San Diego Eye Bank (San Diego, CA, USA) is authorized for organ and/or tissue donation for research in accordance with the California‘s Uniform Anatomical Gift Act effective since 10-Aug-2020. For the present study, 14 lenses from seven unidentifiable donors aged 20 to 30 years were supplied from 30-Nov-2024 to 30-Mar-2025. Ethics Committee approval was not required because lenses are not usable for transplantation or clinical application, would have been discarded, and the present study only involved optical measurements.

1. If you prospectively recruited human participants for the study – for example, you conducted a clinical trial, distributed questionnaires, or obtained tissues, data or samples for the purposes of this study, please report in the Methods:
   1. the day, month and year of the **start and end** of the recruitment period for this study.
   2. whether participants provided informed consent, and if so, what type was obtained (for instance, written or verbal, and if verbal, how it was documented and witnessed). If your study included minors, state whether you obtained consent from parents or guardians. If the need for consent was waived by the ethics committee, please include this information.

Please state the line number(s) in the Methods where this is reported **123 - 129**

**X** Completed ___ N/A

1. If you are reporting a retrospective study of, for example, medical records, archived samples, survey data, please report in the Methods section:
2. the day, month and year when the data were accessed for research purposes
3. whether authors had access to information that could identify individual participants during or after data collection

Please state the line number(s) in the Methods where this is reported ______

___ Completed **X** N/A
